# Analysis of School-Level Vaccination Rates by Race, Ethnicity, and Geography in New York City

**DOI:** 10.1001/jamanetworkopen.2022.31849

**Published:** 2022-09-15

**Authors:** Brian Elbel, Geng Eric Zhou, David C. Lee, Willy Chen, Sophia E. Day, Kevin J. Konty, Amy Ellen Schwartz

**Affiliations:** 1 Department of Population Health, New York University Grossman School of Medicine, New York; 2 Wagner Graduate School of Public Service, New York University, New York; 3 Department of Emergency Medicine, New York University Grossman School of Medicine, New York; 4 Maxwell School, Syracuse University, Syracuse, New York; 5 Bureau of School Health, New York City Department of Health and Mental Hygiene, New York

## Abstract

This cross-sectional study of New York City school data examines differences in COVID-19 vaccination rates by race, ethnicity, and borough.

## Introduction

Vaccinations for COVID-19 have proven safe and effective for preventing serious illness for most children.^1,2^ Race, ethnicity, and geography are important correlates of vaccination rates for adults, while data have proven limited in understanding whether this is true for children.^3^ In this cross-sectional study, we took advantage of newly released public school–level vaccination rates in New York City (NYC), where geographically distributed schools with varying racial and ethnic concentration can shed important light on differences by race, ethnicity, and geography for COVID-19 vaccination rates for children.

## Methods

NYC recently posted school-level vaccination data gathered from the NYC Department of Health and Mental Hygiene’s centralized Citywide Immunization Registry.^4^ These data included school-level percentages of students fully (not including boosters) or partially vaccinated for children ages 5 years and older. Using a consistent school identifier, we matched the March 9, 2022, data release to school-level data from the 2020-2021 School Report card.^5^ To correspond with the differential timing of vaccination approval for children older vs younger than age 12 years, we categorized schools into 3 groups: elementary (serving grades pre-kindergarten to 5, younger than age 12 years), middle-high (grades 6 to 12, likely older than 12 years), and other grades (eg, schools with grades kindergarten to 8, kindergarten to 12).

We created a set of mutually exclusive and exhaustive categorical variables identifying the majority race or ethnicity of students in each school (Hispanic, Black, White, Asian, or no majority race). We then used ordinary least squares (weighted by share of total enrollment) to determine the regression-adjusted association between school-level vaccination and race and ethnicity, both overall and in a model with race and ethnicity by borough interactions. We also controlled for school-level percentage of students that were female, economically disadvantaged, experiencing homelessness, or in foster care. We used the margins command in Stata version 17 (StataCorp) to show the regression-adjusted vaccination percentage.

This research was reviewed by the NYU Langone institutional review board and was determined to meet the criteria for exemption 4 under 45 CFR 46. This study followed the STROBE reporting guideline.

## Results

Among the 1574 schools not exclusively serving special education students, schools had a weighted mean average of 979.7 students (95% CI, 862.2-1097.2); weighted averages of student populations were 48.2% female (95% CI, 47.7%-48.7%), 22.9% with disability (95% CI, 22.1%-23.8%), 75.0% economically disadvantaged (95% CI, 73.9%-76.1%), and 8.4% experiencing homelessness (95% CI, 8.0%-8.7%). Two-thirds of the schools had a majority race or ethnicity in their student population, ranging from 35.6% Hispanic (95% CI, 32.9%-38.2%) to 7.5% White (95% CI, 5.8%-9.1%) (Table). Across NYC, the average school-level vaccination percentage was 52.7% (95% CI, 51.4%-54.1%).

**Table.** School-level Descriptive Statistics Weighted by School Enrollment, NYC Public School Students 2020-2021 AY

Characteristics	Weighted % (95% CI)
No. of total students, mean (95% CI)	979.7 (862.2-1097.2)
At least first dose	60.2 (59-61.4)
Fully vaccinated	52.7 (51.4-54.1)
Sex	
Boys	51.8 (51.3-52.3)
Girls	48.2 (47.7-48.7)
Student with disability	22.9 (22.1-23.8)
Economically disadvantaged students	75.0 (73.9-76.1)
Experiencing homelessness	8.4 (8.0-8.7)
Students in foster care	0.5 (0.5-0.6)
Borough of schools	
Bronx	19.8 (18.6-21.1)
Brooklyn	29.1 (26.9-31.3)
Manhattan	14.8 (13.5-16.0)
Queens	29.5 (27.6-31.5)
Staten Island	6.8 (5.5-8.0)
School-level majority race or ethnicity	
Asian	11 (8.5-13.6)
Black	12.7 (11.2-14.3)
Hispanic	35.6 (32.9-38.2)
White	7.5 (5.8-9.1)
Not in any majority	33.2 (29.9-36.5)
Mean race or ethnicity	
Asian	18.7 (17.3-20.2)
Black	21.0 (19.8-22.2)
Hispanic	41.3 (39.8-42.7)
White	16.0 (14.8-17.2)
Other^a^	3.0 (2.8-3.2)
School type^b^	
Elementary	36.5 (33.6-39.4)
Middle and above	51.4 (48.1-54.6)
Other grade span	12.1 (10.2-14.0)

Abbreviations: AY, academic year; NYC, New York City.

^a^ Other race includes multiracial and Native American in city school-level data.

^b^ School type corresponds to the following categories: elementary for grades kindergarten through 5, with likely students under the age of 12 years; middle and above for grades 6 through 12, with students over the age of 12 years; other grade span for grades kindergarten to 8 or kindergarten through 12.

In our overall model, schools with a majority of Asian students had the highest vaccination rate (66.2%; 95% CI, 63.9%-68.5%), with a majority of Hispanic students in the middle (53.5%; 95% CI, 52.4%-54.6%), and a majority of Black (44.0%; 95% CI, 42.8%-45.3%) and White (44.0%; 95% CI, 39.9%-48.1%) students having the lowest rates (Figure, panel A). Schools categorized as middle-high had higher vaccination rates (64.9%; 95% CI, 64.0%-65.7%) than elementary (38.8%; 95% CI, 37.9%-39.8%). Vaccination rates varied by borough, ranging from the highest in Manhattan (59.7%; 95% CI, 58.2%-61.1%) to the lowest in Staten Island (38.6%; 95% CI, 35.2%-41.9%).

**Figure.**
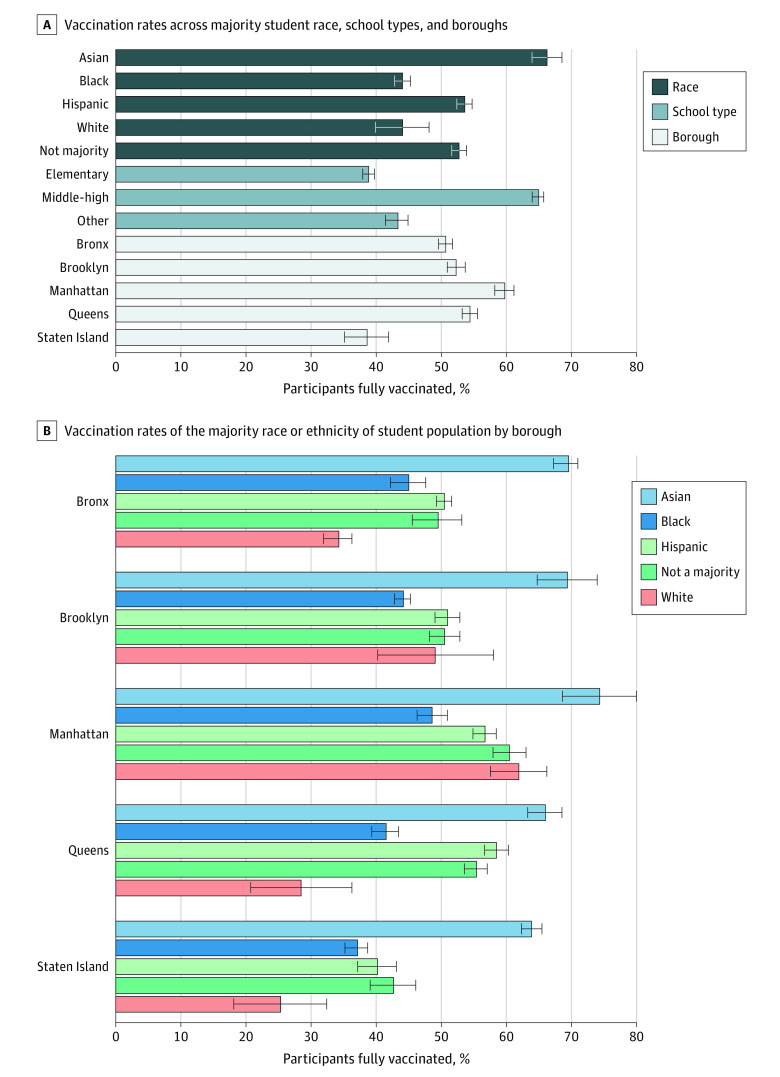
School-Level Fully Vaccinated Rates by Race, Ethnicity, and Geography Vaccination rates refer to the percentages of fully vaccinated students and are projected from the regression analysis. Error bars indicate 95% CIs.

Differences in race and ethnicity by borough were also present in the model with a race and ethnicity by borough interaction (Figure, panel B). While schools with a majority of Asian students consistently had the highest vaccination percentage regardless of borough, other differences by borough were apparent. Schools with a majority of White students in Manhattan (61.9%; 95% CI, 57.6%-66.2%) and Brooklyn (49.1%; 95% CI, 40.2%-58.0%) had a higher school-level vaccination percentage than schools in the Bronx (34.1%; 95% CI, 32.0%-36.3%), Queens (28.5%; 95% CI, 20.8%-36.3%), or Staten Island (25.4%; 95% CI, 18.2%-32.6%).

## Discussion

We found key differences in vaccination rates by race and ethnicity of student population among schools in NYC, with schools with a majority of Asian students and schools in Manhattan having the highest vaccination rates. Some of these differences are similar to adult data (ie, Black vs White) and the aggregated unadjusted city-level data^6^ on children. Differences also emerged by geography, both overall and by race and ethnicity, as has been shown in state-level data.^3^ This information could be used to target additional policies and programs. Study limitations included a school-level approach, limited control variables, and possible missing data on vaccinations administered outside of NYC.

Future work must examine these trends using individual-level data, including personal attitudes and vaccine hesitancy. We must also further examine the social, structural, and political factors that influence vaccination rates among children and consider what policies or program could address this gap.
